# Structure and function of neurovascular unit in arterial hypertension

**DOI:** 10.3389/fnmol.2025.1714892

**Published:** 2026-01-12

**Authors:** Ewa Kozniewska, Marta Aleksandrowicz

**Affiliations:** Laboratory of Preclinical Research and Environmental Agents, Mossakowski Medical Research Institute, Polish Academy of Sciences, Warsaw, Poland

**Keywords:** astrocytes, endothelial glycocalyx, endothelium, glymphatic system, hypertension, neurovascular coupling, pericytes

## Abstract

Arterial hypertension is considered a main risk factor for cognitive impairment and stroke. Although chronic hypertension leads to adaptive changes in the lager cerebral blood vessels which should protect the downstream microvessels, profound changes in the structure and function of cerebral microcirculation were reported in this disease. The structural changes lead to dysregulation of the neurovascular unit and manifest themselves in particular as endothelial dysfunction, disruption of the blood-brain barrier and impairment of neurovascular coupling. The impairment of neurovascular coupling results in inadequate functional hyperemia, which in turn may lead to cognitive decline and dementia. In this review the effects of chronic arterial hypertension on the essential components of neurovascular unit involved in neurovascular coupling such as endothelial cells, astrocytes and pericytes are discussed.

## 1 Introduction

The brain is entirely dependent on the blood supply due to the high rate of oxygen and glucose metabolism and lack of the energy reserves. The human brain receives as much as 15% of the cardiac output, although it weighs only 2% of the body weight (Williams and Leggett, 1989). The mean cerebral blood flow does not change during local alterations of brain activity, but a redistribution of flow takes place to deliver more blood to metabolically activated areas thanks to the efficient local mechanisms of microflow regulation.

The local regulation of cerebral blood flow was first suggested in 1890 by Roy and Sherrington (1890). Performing the pioneering experiments in which they stimulated the sciatic nerve in dogs and measured changes of blood volume in the brain, they came to the conclusion that “the brain possesses an intrinsic mechanism by which its vascular supply can be varied locally in correspondence with local variations of functional activity.” About 90 years later, Larsen et al. (1978) and Lassen et al. (1978) published for the first time a functional mapping of the human brain, detecting activated brain regions by their increases in blood flow.

Extensive experimental studies on the morphological basis and on the mechanisms of this regulation have resulted in the introduction of two new concepts–the neurovascular unit and the neurovascular coupling (Iadecola, 2017; Nippert et al., 2018).

The neurovascular unit (NVU) is a structural and functional entity composed of neurons, astrocytes, and the microvascular endothelium, which together with perivascular astrocytic foot processes, pericytes and the extracellular matrix, form the blood-brain barrier (BBB).

In recent years, the participation of the luminal lining of the endothelial cells–the glycocalyx–in the BBB mechanisms is extensively discussed (Zhao et al., 2021).

The BBB is one of the primary mechanisms of the central nervous system (CNS) homeostasis. The maintenance of chemical composition of the extracellular fluid ensures the stabilization of resting potentials and the proper excitability of nerve cells. The presence of the blood-brain barrier also helps to maintain a constant volume of the extracellular space and thus the stable intracranial pressure. Keeping ions and proteins inside capillaries produces a gradient of osmotic pressure between the intra- and extravascular spaces, so that water that has been filtered through the wall of the capillary system is resorbed into the vascular system. This is essential for the pressure-volume balance of the brain, which does not have true lymphatic vessels and, together with the vascular system and cerebrospinal fluid, is surrounded by a rigid skull. However, the equivalent of the lymphatics, named a glymphatic system, has been described in the CNS (Iliff et al., 2012). This pathway will be discussed in more detail later in this review.

The structural components of the NVU are closely and reciprocally linked to each other to ensure an efficient system of microflow control (Abbott et al., 2006; Iadecola, 2017). In brief, the signals generated by activated neurons induce vasodilation of precapillary arterioles and increase microflow in a process named neurovascular coupling (NVC), which involves coordinated action of all elements of the NVU (Iadecola, 2017). The NVC is essential for the normal functioning of the brain. Impairment of neurovascular coupling is associated with cognitive decline and dementia as reported in neurodegenerative diseases and in hypertension (Iadecola and Gottesman, 2019; Meissner, 2016; Santisteban et al., 2023; Yu et al., 2020). According to Santisteban et al. (2023) long lasting high arterial blood pressure disturbs the structure and function of NVU, which in turn may result in the impairment of the NVC.

In this review, the effects of chronic arterial hypertension on the essential components of neurovascular unit involved in neurovascular coupling such as endothelial cells, astrocytes and pericytes are discussed.

The discussion of the impact of hypertension on the structure and function of the NVU is preceded by a brief description of the physiology of individual NVU components. Although the complex nature of the NVU in health has been extensively reviewed (Lochhead et al., 2020; McConnell and Mishra, 2022; Presa et al., 2020; Segarra et al., 2019), the present review is supplemented with recent data on the important role of the endothelial glycocalyx and the glymphatic system in NVU homeostasis.

This review is based on the findings from the experimental models of human arterial hypertension such as: (1) pharmacological models [Angiotensin II (ANG II)-, deoxycorticosterone acetate (DOCA)- salt-, high salt diet (HSD)-, and N-nitro-L-arginine methyl ester (L-NAME)-induced hypertension]; (2) genetic models of essential hypertension [spontaneously hypertensive rat (SHR); stroke-prone SHR; Dahl salt-sensitive rats]; and (3) surgically induced hypertension [the two-kidney one-clip model (i.e., constriction of only one renal artery), the two-kidney two-clip model (i.e., aortic constriction or constriction of both renal arteries), or the one-kidney one-clip model (i.e., constriction of one renal artery and ablation of the contralateral kidney)].

It should be mentioned that in recent years, thanks to modern genomics and omics technologies offering cell-specific molecular insights (such as e.g., single-cell RNA sequencing), considerable heterogeneity of the cerebrovascular network was demonstrated depending on the brain region or the position within the vascular tree.

For the readers interested in the issue of segmental heterogeneity and in the evolving concept of neurovascular complex, we recommend recent reviews by Schaeffer and Iadecola (2021), Matsuoka et al. (2022) and Iadecola et al. (2023).

## 2 Physiology of the NVU

### 2.1 Cerebral microvascular endothelium and glycocalyx

**FIGURE 1:**
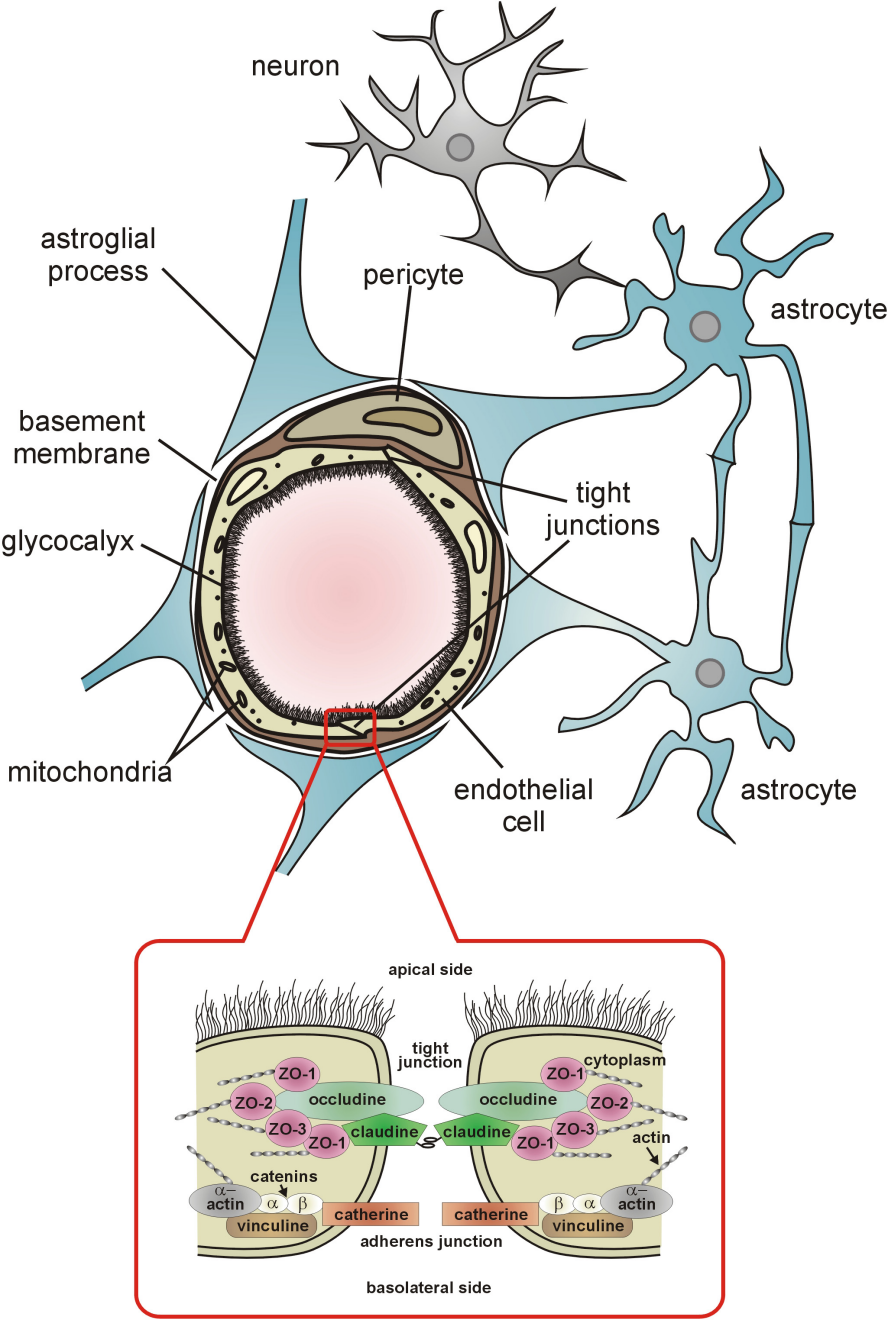
The components of the blood-brain barrier. Detailed description is provided in the text.

The endothelial cells (ECs) of capillaries form the structural basis of the BBB. They are connected by tight and adherens junctions (Figure 1) that restrict paracellular transport to the brain of unwanted and unnecessary blood born molecules and ions (Abbott et al., 2006; Daneman and Prat, 2015). The tightness of tight junctions (TJs) in the BBB is evidenced by their high electrical resistance which approximates 2,000 Ω cm^–2^. In other organs with continuous capillary wall (e.g., skeletal muscles) the resistance of the intercellular junctions does not exceed 30 Ω cm^–2^ (Butt et al., 1990). In addition, endothelial cells of cerebral capillaries under normal conditions have very few cytoplasmic vesicles that limits transcytosis (Langen et al., 2019). Delivery of essential, not freely diffusing nutrients is based on specialized transport systems (Abbott et al., 2006; Daneman and Prat, 2015; Langen et al., 2019).

The luminal surface of the cerebral capillary endothelium is lined with a hundreds of nanometer thick glycocalyx (eGC) synthesized by the endothelial cells (Zhao et al., 2021; Figure 1). It is composed mainly of transmembrane and membrane-bound proteoglycans, glycosaminoglycans, and glycoproteins, predominantly hyaluronan, syndecan-1, and heparan sulfate (Reitsma et al., 2007). The eGC is best known as a sensor and transducer of shear stress forces exerted on the endothelium by circulating blood and intravascular pressure (Weinbaum et al., 2003; Tarbell and Pahakis, 2006; Curry and Adamson, 2012) and plays a key role in shear stress activation of the endothelial production of NO and prostacyclin (Resnick and Gimbrone, 1995; Chien et al., 1998; Andrews et al., 2014; Williams and Flood, 2015). Both compounds are vasodilators and anticoagulants. Endothelial NO is essential for the maintaining resting cerebral blood flow and microvascular resistance, whereas prostacyclin ensures patency of microvessels by inhibiting intravascular coagulation (Gryglewski et al., 1997; Iadecola et al., 1994; Kozniewska et al., 1992).

Recent data indicates that glycocalyx is essential also for the maintenance of the barrier properties of the capillary endothelium (Kutuzov et al., 2018; Stoddart et al., 2022; Zhao et al., 2021; Zhu et al., 2018). It has been demonstrated that enzymatic degradation of the eGC under physiological conditions led to increased vesicular activity in the endothelium, transcytosis, and subsequent BBB leakage (Zhu et al., 2018). It has been also shown with two-photon microscopy imaging of single cortical capillaries in anesthetized mice that glycocalyx forms a barrier to large but not to small molecules (Kutuzov et al., 2018).

It is worth mentioning that luminal portion of eGC is negatively charged and is responsible for repelling from the vessel wall the negatively charged albumins and inflammatory leukocytes (Foote et al., 2022). Thus, under physiological conditions the eGC stabilizes BBB, prevents leakage of plasma components and adhesion of leukocytes to the endothelium, inhibits platelet activation and intravascular coagulation.

The abluminal side of the cerebral vessels endothelium is surrounded by a basement membrane shared with the adjacent pericytes (Figure 1). Capillary pericytes and ECs are in almost 98% covered by astrocytic foot processes (end-feet).

The interaction between the cells constituting the NVU is essential for the formation and maintenance of the BBB as well as for the adequate blood supply of the neurons.

To maintain selective BBB permeability, the endothelium regulates the exchange of fluids and solutes, including plasma proteins by paracellular and transcellular transport. The paracellular transport is characterized by passive diffusion for solutes, leading to relatively unregulated movement of substances compared to the more tightly controlled transcellular pathway (Abbott et al., 2006; Komarova and Malik, 2010). The paracellular transport is limited by the above-mentioned tight junctions and adherens junctions, which connect adjacent endothelial cells into the monolayer to limit the transport of plasma proteins from the vessel lumen to stroma (Komarova and Malik, 2010; Figure 1).

The TJs are composed of three integral membrane proteins: claudins, occludin, and the adhesion proteins (Figure 1). Claudins are considered to establish the backbone of TJs (Günzel and Yu, 2013). The claudin molecules of adjacent cells are linked by adhesion proteins. Both claudins and occludin appear to regulate the tightness of TJs, depending on the level of phosphorylation (Hirase et al., 1997; Yamamoto et al., 2008; Ma et al., 2017). Some experimental data indicate that claudin-5, the major claudin of the BBB, is able to induce barrier function in rat brain endothelial cells (Ohtsuki et al., 2007) whereas claudin-5 deficiency is associated with increased BBB permeability (Nitta et al., 2003). On the other hand, phosphorylation of occludin has been reported to stabilize tight junction, its electrical resistance and permeability (Farshori and Kachar, 1999; Tsukamoto and Nigam, 1999; Wachtel et al., 1999). The intracellular proteins zonula occludens: ZO-1, ZO-2 and ZO-3 link claudins and occludin with actin of the cytoskeleton. *In vitro* studies have shown that zonula occludens, particularly ZO-1, similarly to occludin, is a tight junction regulatory protein (Huber et al., 2001). The most important intracellular signals influencing tight junction formation and permeability include protein kinase C, adenylate cyclase, and calcium ions (Gloor et al., 2001). The adherens junction, composed of vascular endothelial (VE) cadherin, also contacts cytoskeletal actin by means of the corresponding catenin subunit, and seems necessary for the formation and stabilization of tight junction (Huber et al., 2001). *In vitro* studies have shown that modification of the cadherin molecule leads to a reduction in the tightness of TJs (Navarro et al., 1995; Mitic and Anderson, 1998). The barrier function of TJs also significantly depends on the spatial structure of actin. The numerous experimental studies have shown that reorganization of this cytoskeleton protein leads to the destabilization and reduction of tightness of TJs (Huber et al., 2001; Kniesel and Wolburg, 2000). The reorganization of cytoskeletal actin may be one of the causes of increased TJs permeability in hypertension (Nag, 1995).

The second type of endothelial transport, named transcellular pathway or transcytosis, is responsible for the transport of macromolecules across the endothelial barrier (Bourdet et al., 2006). Transcytosis is typically energy-dependent and allows for the selective transport of larger or charged molecules that cannot diffuse freely through tight junctions. It also maintains transendothelial oncotic pressure (Komarova and Malik, 2010). Transcytosis involves the fission of caveolin-1 (Cav-1)-enriched plasma membrane macrodomains, from the endothelial cell’s luminal surface, and then the movement of caveolar vesicles to the basal surface. The vesicles release macromolecules through exocytosis after fusing with the abluminal side’s plasma membrane (Minshall et al., 2002). Currently it is postulated that endothelial transcytosis can be divided into: non-selective adsorptive transcytosis, in which charged interactions between the molecules and the plasma membrane facilitate cargo entry, and receptor-mediated transcytosis (RMT), in which ligand–receptor binding initiates endocytosis (Yang et al., 2025). Recently, RMT is believed to be a potential route for delivering drugs with high molecular weights to the brain (Baghirov, 2025; Zhang et al., 2025). In physiological conditions, endothelial cells usually exhibit a low level of transcytosis (Ayloo and Gu, 2019). Its increased activity is rather associated with pathological conditions (Knowland et al., 2014; Yang et al., 2020). Despite that, both transcytosis and paracellular transport play an important role in maintaining tissue fluid homeostasis, however they act through different mechanisms, contribute differently to physiological processes and responses to pathological conditions.

**FIGURE 2:**
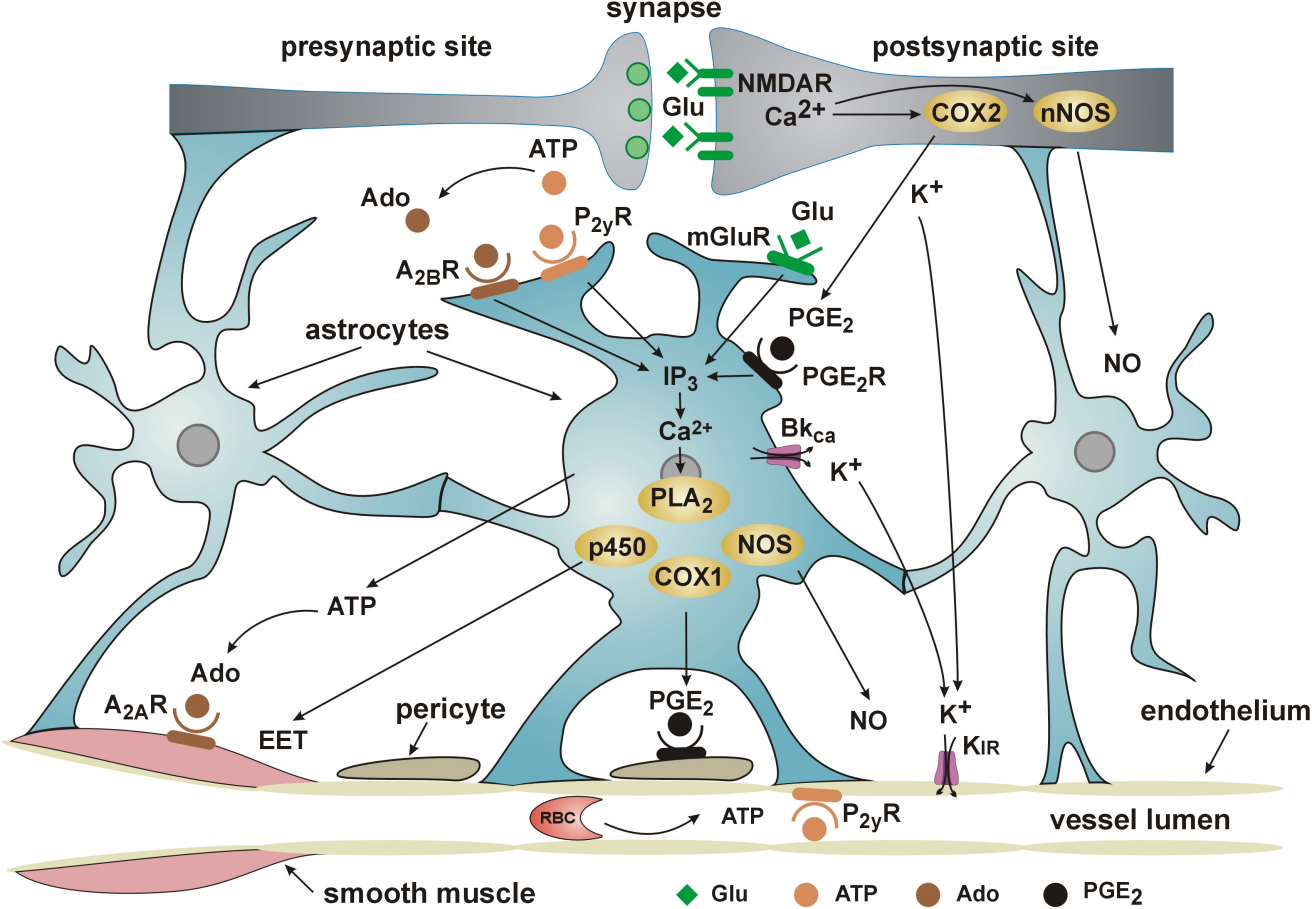
Current view of the cooperation of NVU elements in functional hyperemia/neurovascular coupling. Detailed description of the role of the endothelium in NVC is provided in the text. ADO, adenosine diphosphate; ATP, adenosine triphosphate; A_2*A*_R and A_2*B*_R, adenosine receptors; COX1 and COX2, cyclooxygenases 1 and 2; p450, cytochrome P450; EET, epoxyeicosatrienoic acid; IP3, inositol trisphosphate; PGE_2_, prostaglandin E_2_; PGE_2_R, prostaglandin E_2_ receptor; K_IR_, inward rectifier potassium channels; BK_Ca_, channel, large conductance calcium-activated potassium channel; Glu, glutamate; mGluR, metabotropic glutamate receptor; NMDAR, N-methyl-D-aspartate receptor; NO, nitric oxide; NOS, nitric oxide synthase; nNOS, neuronal nitric oxide synthase; P_2*Y*_R, purinoceptor; PLA2, phospholipase A2; PLD2, phospholipase D2.

Although sealing in the BBB is considered to be the most important function of the cerebral microvascular endothelium, the current knowledge points out the role of the endothelium in the initiation and propagation of the signals underlying NVC and functional hyperemia (Chen et al., 2014; Mishra et al., 2016; Iadecola, 2017; Longden et al., 2017; Rungta et al., 2018; Santisteban et al., 2023). According to a current point of view, increased extracellular concentration of K^+^, associated with neural firing (Figure 2), activates inward-rectifier potassium channels Kir2.1 in capillary endothelial cells which leads to endothelial hyperpolarization. The hyperpolarizing current back-propagates along endothelial cells and spreads to vascular smooth muscle cells in precapillary arterioles through the myo-endothelial junctions to cause dilation of upstream penetrating arterioles and pial arteries, thereby inducing an increase in blood flow to the site of signal initiation (Longden et al., 2017; Rungta et al., 2018).

### 2.2 The role of astrocytes

The astrocytes spreading between the neurons and capillary wall (Figures 1, 2), cover with their extensive foot processes (end-feet) about 98% of the abluminal surface of the endothelium. They are essential for the formation and maintenance of the BBB as discussed by Abbott et al. (2006).

Astrocytes are also important mediators of functional hyperemia (Figure 2). For a long time they were considered as a main source of the signals responsible for NVC (for review see Lia et al., 2023). It has been established that during the release of glutamate from the firing neurons, astrocytic glutamate receptors, both metabotropic and NMDA, are stimulated what results in the increase of intracellular Ca^2+^ concentration. The astrocytic Ca^2+^ concentration increases also due to the stimulation of purinergic (P_2*y*_R, A_2*B*_R) and prostaglandin E_2_ (PGE_2_R) receptors. Activation of calcium-dependent metabolic pathways in astrocytes results in the release of vasodilators such as epoxyeicosatrienoic acid (EET), prostaglandin PGE_2_ and nitric oxide (NO). EET inhibits vascular smooth muscle tone directly whereas PGE_2_ seems to diminish capillary resistance indirectly by hyperpolarizing the pericytes (Lia et al., 2023). The main action of astrocytic NO is it to inhibit production by astrocytes of 20-hydroxyeicosatetraenoic acid (20-HETE), a product of omega-hydroxylation of arachidonic acid, and a strong vasoconstrictor (Hoopes et al., 2015).

Astrocytes also participate in the exchange of cerebrospinal (CSF) and interstitial fluid (ICF), of the brain parenchyma which helps to eliminate metabolic waste products from the central nervous system. This clearance mechanism, known as glymphatic system, is mediated by a water channel aquaporin-4 (AQP4) located at the astrocytic end-feet processes close to the capillary wall (Iliff et al., 2012). According to the glymphatic theory, the CSF flows along the periarterial space, passes through AQP4 channel and enters the parenchyma where it mixes with ISF, and leaves the brain along perivenous space. This way, waste products included in ISF are cleared out of the brain. It has been demonstrated that lack of AQP4 at the gliovascular interface impairs clearance and transport via the glymphatic system (Iliff et al., 2012).

### 2.3 The pericyte

Pericytes are pluripotent, perivascular mural cells found in precapillary arterioles, capillaries and postcapillary venules. The density of pericytes covering brain capillary wall varies between 30 and 99% of the abluminal surface (Dalkara et al., 2011; Hartmann et al., 2015). They are embedded in the basement membrane of the endothelial cells (Sims, 1991; Mathiisen et al., 2010). Although the ratio of pericytes to endothelial cells in cerebral capillaries is 1:3, the pericytes send out the extensive projections along the capillaries (Sá-Pereira et al., 2012). The processes of adjacent pericytes do not overlap, although they end in close proximity. Such a morphology allows for a better contact of pericytes with endothelial cells and astrocytic end-feet. Pericytes are characterized by significant plasticity. Interestingly, it has been demonstrated that elimination of a single pericyte in a mouse cerebral cortex results in the mobilization of the neighboring pericytes to send their projections to the uncovered endothelium (Berthiaume et al., 2018).

Pericytes were reported to control protein expression of the tight junctions (Armulik et al., 2010; Bell et al., 2010; Daneman et al., 2010). Pericyte ablation has been shown to breakdown of the BBB in experimental conditions (Nikolakopoulou et al., 2019).

Pericytes also play a key role in the formation and stabilization of new blood vessels (Durham et al., 2014; Blocki et al., 2018).

Being located between astrocytic end-feet and endothelial cells (Figure 1), pericytes are in excellent position to regulate the resistance to flow through capillaries, particularly as they express the contractile protein alpha-smooth muscle actin (Nehls and Drenckhahn, 1991; Bandopadhyay et al., 2001; Alarcon-Martinez et al., 2018) and receptors for vasoactive compounds such as ATP, noradrenaline, thromboxane A_2_, acetylcholine, PGE_2_ (Fernández-Klett et al., 2010; Peppiatt et al., 2006; Hamilton et al., 2010). Accordingly, numerous studies have shown that pericytes regulate capillary diameter and are important players in NVC (Peppiatt et al., 2006; Yemisci et al., 2009; Hamilton et al., 2010; Hall et al., 2014; Khennouf et al., 2018). As demonstrated by Khennouf et al. (2018) capillaries dilate during activation of the barrel cortex in mice (whisker stimulation) due to the decrease of calcium signal in the pericytes. According to Hall et al. (2014) during functional hyperemia, pericytes may dilate capillaries via a prostaglandin E_2_-dependent pathway as presented in Figure 2.

Some experimental studies reported that ablation or degeneration of pericytes leads to neurovascular uncoupling, reduced oxygen supply to the brain and metabolic stress. Neurovascular deficits lead over time to impaired neuronal excitability and neurodegenerative changes (Kisler et al., 2017, 2020).

## 3 Neurovascular coupling

The increase in microflow adequate to support neuronal activity requires, as mentioned, the close cooperation of all elements of the NVU (Figure 2). The trigger of the flow response is the release of the excitatory neurotransmitter glutamate and stimulation of NMDA and metabotropic glutamate receptors on neurons and astrocytes. The resulting increase in the intracellular Ca^2+^ concentration leads to the release of vasodilators such as PGE_2_, NO and EET. An essential role in NVC is played by K^+^ ions, released during depolarization of neurons and from astrocytic end-feet due to the opening of BK_Ca_ potassium channels. Increasing the extracellular K^+^ concentration activates Kir channels in the endothelial cells, leading to their hyperpolarization. This in turn is transmitted in a retrograde fashion as wave of hyperpolarization to the level of arterioles to dilate them and ensure the increase in blood supply of the activated neurons.

In conclusion, all components of the NVU are essential for the maintenance of the blood-brain barrier and a close cooperation between them is required to ensure proper NVC.

## 4 Effects of arterial hypertension on the components of the neurovascular unit

The arterial hypertension has profound effects on the structure and function of all components of the NVU, resulting in an impaired communication between neurons and microvessels (Calcinaghi et al., 2013; Capone et al., 2012; Girouard et al., 2007; Kazama et al., 2003), neurons and astrocytes (Tagami et al., 1991), and astrocytes and microvessels (Diaz et al., 2019; Marins et al., 2017). The diseased communication, in turn, leads to the impairment of NVC and inadequate functional hyperemia, resulting in a cognitive decline and hypertension-associated dementia (Dahlöf, 2007; Iadecola and Gottesman, 2019). An additional cause of functional impairment associated with hypertension may be the rarefaction of cerebral capillaries, reported in the different animal models of hypertension (Kozniewska et al., 1982; Suzuki et al., 2003; Tarantini et al., 2016) and in the retina of patients with untreated mild hypertension (Bosch et al., 2017). In the context of cognitive deficits, a decreased density of capillaries in the cerebral cortex, as observed in the study by Kozniewska et al. (1982), was associated with a decrease in cerebral microflow.

### 4.1 Impact on the endothelial cells, glycocalyx, and the BBB

Accumulating evidence indicates that the BBB is a dynamic structure which is dysregulated in cardiovascular diseases including hypertension (Lochhead et al., 2020). Hypertension leads to increased endothelial permeability and BBB disruption, which has been shown in several experimental models of hypertension, including: SHR (Tagami et al., 1991; Ueno et al., 2004), Dahl-salt sensitive rats (Pelisch et al., 2011; Maeda et al., 2021), the two-kidney two-clip model of hypertension (Mohammadi and Dehghani, 2014), and angiotensin II-induced hypertension (Vital et al., 2010; Santisteban et al., 2020). Hypertension-induced changes in the BBB comprise unsealing of the TJs, damage of the glycocalyx and endothelial dysfunction. The effect of hypertension on paracellular transport appears complex. Some studies demonstrated dysfunction of paracellular transport (Pelisch et al., 2011, 2013; Biancardi et al., 2014; Mohammadi and Dehghani, 2014), while other indicated mainly a transcellular transport as the determinant of increased capillary leakage (Fragas et al., 2021; Candido et al., 2023). For example Santisteban et al. (2020) showed that sustained elevations in blood pressure caused by ANG II induce morphological and molecular remodeling of TJs. ANG II-dependent decreased expression of mRNA for TJs proteins was observed in Dahl salt-sensitive rats fed a high-salt diet (Pelisch et al., 2011). On the other hand, Fleegal-DeMotta et al. (2009) demonstrated that ANG II did not affect the expression of markers of endothelial tight junctions, including occludin and claudin-5. In contrast, expression of claudin-5 was reduced in stroke-prone SHR (Bailey et al., 2011) and the two-kidney, two-clip model of hypertension (Mohammadi and Dehghani, 2014), however, the role of ANG II was not investigated in these studies. Unlike TJs, there are few experimental studies on the effect of hypertension on VE-cadherin. Recent clinical research showed decreased plasma VE-cadherin levels in hypertensive patients (Tjili et al., 2025). Some data indicate that not paracellular transport but transcytosis is the primary mechanism underlying increased BBB permeability in hypertension, at least in brain areas related to the autonomic system (Fragas et al., 2021; Candido et al., 2023). Fragas et al. (2021), analyzing changes in caveolin-1 expression within hypothalamus of the SHRs, showed increased transcytosis and a positive effect of exercise training on normalization of this type of endothelial transport (Fragas et al., 2021). The recent studies showed an increase in transcellular transport and a reduction in claudin-5 expression in the two-kidney, one-clip model of hypertension (Perego et al., 2025). These latest research suggests that both types of endothelial transport may coexist and may be affected concomitantly in hypertension.

In addition to the negative impact of hypertension on endothelial transport, unfavorable changes in the glycocalyx are also observed. The degradation of the microvascular glycocalyx was demonstrated in stroke-resistant and stroke-prone SHR (Ueno et al., 2004) and in ANG II-induced hypertension (Nag, 1984), what inevitably led to dysfunction of the endothelium. In consequence, glycocalyx degradation may lead to leakage of the blood–brain barrier and the formation of brain edema (Zhu et al., 2018).

The endothelial glycocalyx breakdown is followed by up-regulation of endothelial adhesion molecules, such as endothelial selectin (E-selectin), platelet selectin (P-selectin), vascular cell adhesion molecule (VCAM-1), and intercellular adhesion molecules (ICAM-1) (Rabelink et al., 2010). The vast majority of studies regarding the effect of hypertension on the expression of adhesion molecules have been conducted on peripheral vessels. These studies have demonstrated increased expression of adhesion molecules in stroke-prone SHRs (Liu et al., 1996), two-kidney one-clip hypertensive rats (Mai et al., 1996), and in angiotensin II-induced hypertension (Tummala et al., 1999). Furthermore, clinical trials revealed that patients with moderate hypertension had considerably greater circulating levels of both ICAM-1 and VCAM-1 than their normotensive peers of similar age, body composition, and metabolic profile (Desouza et al., 1997). Another clinical investigation demonstrated significant increases in E-selectin, P-selectin, and ICAM-1, as well as a trend toward increased levels of VCAM-1, in hypertension patients compared to controls (Shalia et al., 2009). Notably, there was an increase in the expression of adhesion molecules in cerebral blood vessels (Suzuki et al., 2001). An increased level of adhesion molecules is critical in the progression of thrombosis and stroke (Blankenberg et al., 2003). This is one of the causes that hypertension is regarded as a major risk factor for stroke development (Johansson, 1999; Przykaza, 2021).

Some experimental studies demonstrated that increased endothelial permeability is associated with increased brain ANG II level rather than with blood pressure changes (Pelisch et al., 2011; Faraco et al., 2016). The AT1 receptors on endothelial cells are essential for initiating the increase in BBB permeability. However, experimental studies have shown that the AT1 receptors in perivascular macrophages (PVM), innate immune cells closely associated with cerebral arterioles, were major contributors to the neurovascular dysfunction (Santisteban et al., 2020). In hypertension, ANG II can enter the perivascular space and activate AT1 receptors in PVMs, leading to the production of ROS through the superoxide-producing enzyme NOX2 (Faraco et al., 2016). The downstream mechanisms by which ANG II-induced oxidative stress alters cerebrovascular function involve nitrosative stress and NO depletion (Girouard et al., 2007; De Silva and Faraci, 2013). ANG II-derived superoxide anion reacts with NO to form a powerful oxidant, peroxynitrite, which, in turn, mediates damage to the endothelium (Beckman et al., 1990). Impaired response of microvessels to endothelium-dependent acetylcholine was observed not only in ANG II-induced hypertension (Girouard et al., 2007) but also in DOCA-salt–induced hypertension (Matin et al., 2016) and in SHR (Freitas et al., 2017).

BBB dysfunction facilitates the infiltration of plasma components into the brain and the production of pro-inflammatory signals that lead to the activation of microglia and astrocytes (Presa et al., 2020). Circulating inflammatory molecules, such as tumor necrosis factor-α (TNF-α), C-reactive protein, interleukin IL-6, and IL-1β, are upregulated in the brain both in hypertensive patients and in hypertensive animal models (Coffman, 2011; Winklewski et al., 2015). Inflammatory factors, in turn, impair endothelial function, resulting in a further reduction in the functionality of the microvasculature (Meissner, 2016). It is known that ANG II–induced hypertension attenuates the increase in neocortex CBF produced by whisker stimulation (Kazama et al., 2003; Girouard et al., 2007). Further studies revealed that impaired neurovascular coupling in ANG II–induced hypertension is associated with ROS production in the subfornical organ (SFO), one of the forebrain circumventricular organs responsible for hormonal release and sympathetic activation that drive the elevation in arterial pressure (Capone et al., 2012). The effect of ANG II on neurovascular coupling was blocked by losartan, the antagonist of AT1 receptors (Kazama et al., 2003). In addition, no impairment of neurovascular coupling was found in mice administered phenylephrine to induce a similar degree of hypertension (Kazama et al., 2003; Capone et al., 2012), suggesting that ANG II, rather than the elevation in systemic pressure *per se*, caused the neurovascular dysfunction. On the other hand, neurovascular coupling to whisker stimulation is also impaired in SHR (Calcinaghi et al., 2013), and losartan treatment did not improve functional hyperemia, indicating that the effects of blood pressure elevation on arterial structure, such as the response of microvessels to acetylcholine, cannot be ignored.

In hypertension, the change of endothelial cell phenotype is observed. Instead of promoting vasodilation and anticoagulation, the endothelial cells in hypertension are pro-contractile, pro-inflammatory, and pro-oxidative. As a result of this unfavorable change, cerebral blood microvessels are unable to respond with vasodilation to increased neuronal activity, pointing to the impairment of NVC (Kazama et al., 2003; Girouard et al., 2007; Capone et al., 2012; Calcinaghi et al., 2013). Since the normal endothelium exerts trophic effects on brain cells and contributes to maintaining the health of neurons, glia, and oligodendrocytes (Iadecola and Gottesman, 2019), the dysfunction of endothelial cells in hypertension may affect all components of NVU.

### 4.2 Impact on astrocytes

Although astrocytes establish a functional bridge between brain perfusion and neuronal activity due to their close contact with blood vessels and synapses, there is still a limited understanding of the changes in their structure and function in hypertension. The increasing amount of recent research sheds new light on this issue (Xia et al., 2025; Diaz et al., 2019; Bhat et al., 2018; Gowrisankar and Clark, 2016; Haspula and Clark, 2021; Marins et al., 2017). One of the first studies investigating the influence of hypertension on astrocytes showed that in stroke-prone SHR rats, astrocytes swelled around the capillaries, due to increased endothelial permeability, which in turn led to their fibrosis (Tagami et al., 1991). The dead neurons were detected adjacent to the fibrous astrocytes, suggesting that dysfunction of astrocytes disturbs the neural environment, leading to neuronal death. Further studies have confirmed structural changes in astrocytes, known as astrogliosis, in stroke-prone SHR (Yamagata, 2012) and SHR (Sabbatini et al., 2002; Tomassoni et al., 2004; Marins et al., 2017). Astrogliosis (also termed reactive gliosis) is a process in which normal astrocytes undergo hypertrophy and proliferation in response to brain injury and become reactive astrocytes. Typically, the term astrogliosis refers to neurodegenerative diseases (Lagos-Cabré et al., 2020), however, arterial hypertension, which induces cerebrovascular changes, can also lead to brain damage, neurodegeneration, and vascular dementia. The transformation of quiescent into reactive astrocytes may result in the formation of a glial scar (Tomassoni et al., 2004; Lagos-Cabré et al., 2020). Although reactive gliosis is a normal physiological response that can protect brain cells from further damage, it also has detrimental effects on neuronal survival, by creating a non-permissive environment for axonal repair. In astrogliosis, the expression of several proteins is enhanced, including glial fibrillary acidic protein (GFAP) and inducible nitric oxide synthase (iNOS). Increased GFAP immunoreactivity was detected in astrocytes of SHR rats in the hippocampus (Sabbatini et al., 2002), frontal cortex, occipital cortex, and striatum (Tomassoni et al., 2004), and in the intermediate insular cortex (Marins et al., 2017). Elevated GFAP levels were also detected in hippocampal astrocytes in a 2-kidney-1-clip (Bhat et al., 2018), DOCA (Pietranera et al., 2006), and high-salt diet-induced model of chronic hypertension (Deng et al., 2017). Expression of inducible NO synthase (iNOS) by reactive astrocytes was observed in stroke-prone SHR (Gotoh et al., 1996). The distribution of iNOS^+^ astrocytes colocalized with the brain histological lesions observed in this model, i.e., petechiae and edema, suggesting that NO generation due to iNOS activation may be involved in the development of hypertensive cerebral lesions (Gotoh et al., 1996).

In addition to the increased expression of GFAP and iNOS, astrogliosis also results in the expression of pro-inflammatory cytokines, including tumor necrosis factor α and β, interleukins, and interferons (Chen and Swanson, 2003). These mediators of inflammation impair the glial trophic support of both the vasculature and neurons (Iadecola, 2013), leading to neuronal injury and neurodegeneration (Chen and Swanson, 2003). Upregulated expression of tumor necrosis factor-α (TNF-α), as well as pro-inflammatory interleukin-6 (IL-6) and interleukin-1β (IL-1β), was detected in astrocytes in a high salt diet-induced model of chronic hypertension (Deng et al., 2017). A shift toward pro-inflammatory TNF-α and a decrease in anti-inflammatory interleukin-10 (IL-10) in both cortex and hippocampus was also shown in the 2-kidney-1-clip model of hypertensive rats (Bhat et al., 2018). Different results were presented by Haspula and Clark (2021). These authors reported that not only the mRNA expression of pro-inflammatory IL-1β, but also the mRNA expression of anti-inflammatory IL-10, was significantly elevated in brainstem and cerebellar astrocytes isolated from SHR compared with Wistar astrocytes. A further part of the studies showed that ANG II treatment resulted in an inhibitory effect on IL-10 gene expression in astrocytes from both brain regions of SHR and Wistar rats, as well as an increase in IL-1β gene expression in brainstem astrocytes from both strains (Haspula and Clark, 2021). Similar pro-inflammatory effects of ANG II were described in other research studies (Benicky et al., 2011; Gowrisankar and Clark, 2016; Bhat et al., 2018). Gowrisankar and Clark (2016) demonstrated that ANG II could trigger an increase in IL-6 mRNA and protein expression in astroglial cultures obtained from SHRs and normotensive control Wistar rats. Moreover, ANG II receptor blockers have been shown to limit inflammatory responses in the brain, suggesting a dependence of neuroinflammatory responses on the AT1 receptor (Benicky et al., 2011; Bhat et al., 2018).

Astrocytic activation also results in the increased production of reactive oxygen species (ROS), leading to neuronal injury and neurodegeneration (Chen and Swanson, 2003). There is a wealth of data supporting the role of ROS in hypertension; however, these data are derived from peripheral cell systems, including resistance arteries and the kidneys (Gowrisankar and Clark, 2016). Few experimental studies performed on isolated astrocytes have shown that in the 2-kidney-1-clip model of hypertension, there is an increased activation of NADPH oxidase and ROS production. Further experiments revealed that inhibition of AT1 receptor, independent of its blood pressure-lowering effect, prevents the activation of NADPH oxidase and ROS production (Bhat et al., 2018). Similar results were obtained by studies Gowrisankar and Clark (2016), in which ANG II also induced ROS generation, and there were no significant differences between ROS generation in SHR-derived astrocytes as compared to the Wistar samples, indicating that an excessive production of ROS in the brain plays a crucial role in the pathogenesis of ANG II-dependent hypertension.

Furthermore, several research groups have noted that hypertension alters intracellular astrocyte Ca^2+^ dynamics (Tallant and Higson, 1997; Diaz et al., 2019). Tallant and Higson (1997) showed that in medullary and cerebellar, but not cortical or hypothalamic astrocytes from neonatal mice, ANG II stimulates a PLC/IP3-mediated increase in Ca^2+^ concentration via AT1 receptor as well as PGI_2_ release. A similar observation in the same model of hypertension, but in adult mice, was reported by Diaz et al. (2019). In these studies, increases of spontaneous and myogenic-evoked Ca^2+^ events were associated with enhanced astrocyte TRPV4 channel activity and expression. Moreover, elevated basal astrocyte Ca^2+^ activity was associated with a greater contribution to resting parenchymal arterioles tone (Diaz et al., 2019). Although it is not entirely clear how this contributes to pathogenesis, the authors hypothesized that sustained intracellular Ca^2+^ elevation in astrocytes could trigger or facilitate the transition of these cells to a proinflammatory state.

Hypertension leads also to dysfunction of the glymphatic system. Experimental investigations have shown that the glymphatic system is considerably impaired in SHRs (Xia et al., 2025; Mortensen et al., 2019) and angiotensin II-induced hypertension (Mestre et al., 2018). Mortensen et al. (2019) and Xia et al. (2025) used dynamic contrast-enhanced magnetic resonance imaging to show that SHRs have delayed glymphatic transport relative to normotensive rats (Mortensen et al., 2019; Xia et al., 2025). This impairment of the glymphatic system in hypertension may be associated with changes in arterial pulsatility produced by arterial stiffness, which disturbs the dynamics of cerebrospinal fluid influx (Krings et al., 2025; Iliff et al., 2012). Moreover, Xia et al. (2025) demonstrated reduced AQP4 expression in the brainstem and olfactory bulb of SHRs, which likely leads to a reduction in fluid transport and clearance (Xia et al., 2025; Klostranec et al., 2021).

Impairment of glymphatic transport in hypertension and decreased efficiency in waste clearance mechanisms may potentially lead to the accumulation of neurotoxic substances, such as Aβ, tau, and α-synuclein. This, in turn, is involved in an increased risk of developing neurodegenerative diseases in hypertension (Iliff et al., 2012).

Considering the importance of astrocyte-neuron and astrocyte-vessel communication, structural and functional changes of astrocytes induced by hypertension could have significant adverse effects on the neurovascular unit and brain plasticity.

### 4.3 Impact on pericytes

There are not much data in the literature directly reporting the effects of arterial hypertension on the structure or function of the brain microvascular pericytes. Few studies have found that vascular alterations associated with hypertension, such as up-regulation of vasoconstricting substances and oxidative stress, can lead to enhanced pericyte contractility and, as a result, capillary rarefaction (Goligorsky, 2010; Hirunpattarasilp et al., 2019). Wu et al. (2020) showed that brain microvascular pericytes from SHRs had abnormal levels of several miRNAs compared to the normotensive group. Authors suggested that these pericyte miRNAs could be potential biomarkers or therapeutic targets for hypertension (Wu et al., 2020). The latest research shows that pericyte metabolism shifts toward glycolysis under hypertensive conditions. This adaptation occurs despite the presence of sufficient oxygen, showing that pericytes prioritize rapid energy production during stress in order to survive and maintain vascular integrity (Morton et al., 2025).

The small number of studies regarding the effect of hypertension on pericytes, suggest that functional and structural changes in pericytes in response to increased arterial blood pressure are relatively less understood compared to other components of the neurovascular unit.

## 5 Conclusion

Arterial hypertension has an adverse effect on all components of the neurovascular unit.

The most popular animal models used to study the influence of hypertension on the neurovascular unit include SHRs, stroke-prone SHRs, two-kidney two-clip models, and angiotensin II-induced hypertension. Regardless of the experimental model, the results showed increased endothelial permeability, BBB disruption, glycocalyx damage, an increase in adhesion molecule expression, astrogliosis, changes in intracellular astrocyte Ca^2+^ dynamics, impairment of the glymphatic system and possibly pericyte dysfunction. Furthermore, there is a disruption in communication between neurons, astrocytes, and microvessels. All of these changes ultimately lead to poor neurovascular coupling and insufficient functional hyperemia, which in turn contributes to cognitive decline and hypertension-related dementia.

Understanding the changes occurring in the structure of the neurovascular unit in hypertension should enable the development of antihypertensive therapy with a protective effect on the higher brain functions. Indeed, several studies have linked antihypertensive treatment to decreased cognitive impairment (Iadecola and Gottesman, 2019).
